# Later-life transitions and changes in prescription medication use for pain and depression

**DOI:** 10.1186/s12877-022-02921-y

**Published:** 2022-03-17

**Authors:** Jack Lam, Mike Vuolo

**Affiliations:** 1 Institute for Social Science Research, Life Course Centre – University of Queensland, Level 2, Cycad Building (1018), 80 Meiers Road, Indooroopilly, QLD 4068 Australia; 2 Ohio State University, Columbus, USA

**Keywords:** Health and Retirement Study, substance use, prescription medication, life transitions, fixed-effects models

## Abstract

**Background:**

Over the past two decades, prescription medication use for pain and depression increased dramatically. Most studies consider the early life course, despite a similar increase among those in later life. In this paper, we examine whether and how later life transitions may relate to changes in medication use.

**Methods:**

We draw on data from the Health and Retirement Study and fixed-effects models to examine whether work, family, and civic transitions in later life are related to changes in the usage of prescription pain and depression medication.

**Results:**

Results show that individuals had higher odds of regularly using prescription pain and depression medications in periods when out of the labor market. Higher odds of depression medication use were also associated with periods of widowhood, and lower odds of use when frequently volunteering. Such relations persist adjusting for reported levels of pain and depression.

**Conclusion:**

Our findings call attention to the importance of social ties and the presence of actors that may regulate health behaviors, as well as a change in social context, that may shape medication use in later life.

**Supplementary Information:**

The online version contains supplementary material available at 10.1186/s12877-022-02921-y.

Life course transitions have been of considerable interest to sociologists, permitting an understanding of how individuals and families fare across commonly experienced events [1–4]. A focus on these transitions considers the impact and variation in experiences, providing an understanding of factors such as social support and coping that may be of importance [5, 6]. Studies of the life course can inform policies and practice to assist individuals as they experience different events. Scholars have studied how individuals fare as they enter different social institutions, through transitions into parenthood or marriage, or from school-to-work [1, 7, 8]. In later life, transitions may encompass both *entries* into and *exits* from social institutions, via retirement and widowhood, or through grandparenthood and civic engagement. Studies commonly examine how life events relate to changes in health and wellbeing outcomes [9, 10], while also considering how health and wellbeing may act as antecedents to life course transitions by predicting entries or exits from social institutions or roles [11]. Nevertheless, less empirical research has focused on changes in health behavior, such as medication use, as related to life course transitions.

We aim to address this important gap in the current paper, given psychoactive prescription medication use is increasingly common in later life, as well as important to understand due to recent trends in substance use and misuse [12, 13]. The social context of substance use has changed considerably. On the one hand, substances such as cannabis have been subject to increasing liberalization of attitudes, resulting in legal recreational and medical availability [14]. On the other hand, the overdose epidemic has brought increasing attention to the need to control certain substances. The beginning of the overdose crisis is commonly attributed to a rise in the number of prescription psychoactive substances [15, 16]. Although opioids have deservedly captured national attention, usage of classes of other prescription substances have simultaneously increased, including medications for depression and anxiety such as tranquilizers and sedatives [17, 18].

Substance use and binge drinking have been raised as concerns for older adults [12, 13]. Medication use in later life may be the result of a range of physical and mental ailments that inflict individuals as they age. For instance, pain is a common experience in later life, reported by a majority of older adults [19]. Studies also show a U-shaped curve whereby self-reported depressive symptoms decline in early adulthood but rises again in later life [20, 21]. Strategies to manage pain and depressive symptoms include physician prescribed medications to manage health conditions, though older adults may also be drawn to prescription medications to alleviate experiences related to events such as role loss that may similarly produce the physical experience of pain [22] and depression [20].

Drawing on and extending studies from sociology and gerontology, as well as social control theory [23], we highlight the role of social support and control for health behavior, to investigate the dynamics of social roles and medication use in later life. We hypothesize and empirically test whether these transitions relate to changes in medication use, specifically for depression and pain. Importantly, given the richness of the dataset with self-reported measures of pain and depression, we adjust for these measures to investigate whether above and beyond reported levels of pain and depression, life course transitions have a particular effect in shaping medication use.

## Background

Role transitions denote changes in the social environment and forms of social influence to which individuals may be subjected. Social control theory [24, 25] underscores relationships as an important component in regulating individual behavior, such that those who are more socially isolated may be more likely to engage in risky or deviant behavior as compared to those more socially integrated. At one level, family and work represent two proximate social environments that place controls and constraints on health behaviors [26]. Life course transitions into and away from these environments therefore indicate a change in context, and related social influence and constraints. Within the health literature, a body of evidence supports the expectation that the socializing forces of family and work play key roles in regulating health behaviors, and this may operate through the presence of important actors. The removal or addition of these actors (partner, family members, supervisor, colleague) could therefore relate to changes in substance use [27]. Spouses in particular have been shown to be a powerful socializing force [28], where they sanction and intervene to regulate behavior, engage in or model desired health behaviors, as well as engage in discussions of specific health behaviors that may be of concern [28, 29].

Beyond their controlling or constraining influence, relationships confer social, instrumental, emotional, and informational support. Marriage confers norms around the provision of support, which act to curtail and regulate partner’s unhealthy behavior, as well as through ‘care work’ (i.e. providing care for one another especially in times of distress or change) and ‘health behavior work’ (i.e. any form of activity that enhances and promotes family members’ health behavior) [30]. Employment can also be both intrinsically and extrinsically rewarding in a manner that promotes health [31]. Such forms of support may buffer against stress or pain, which may in turn relate directly and indirectly to lower substance use and misuse.

Social roles also influence how individuals see themselves, and this internalized form of social control may exert influence on medication use [26]. Indeed, the perceived incompatibility of substance use with self-recognition of adult status has been shown to lead to its cessation during early adulthood [32]. Given the legitimate uses and subsequent normalization of prescription medication [33], especially for ailments occurring in later life, the degree to which this would apply to such substances at this point in the life course is not yet clear. Given the demands and responsibilities of being a partner and an employee, however, these social roles may nevertheless be perceived and experienced as incompatible with increased reliance on medication. The loss of these roles could therefore remove such barriers, as one form of deterrent.

At the same time, we note that aging is not a uniform process of decline and the loss of social roles. Entries into new social roles could also denote the appearance of new forms of social influence. Individuals may gain roles through different events, such as the transition to becoming a grandparent [34] or in becoming a volunteer [35]. For example, the transition to grandparenthood has been found to be associated with individual wellbeing, fewer depressive symptoms among women, and increased subjective life expectancy among employed men who become grandfathers [36]. New social roles such as through volunteering may help facilitate social interactions and support [37, 38]. While existing research has found formal volunteering to be associated with improved wellbeing [39], it may have implications for substance use through increased social support via new friendships and interactions.

Employment and volunteering may also provide regularity to a person’s schedule, modulating behavioral and circadian rhythms, with lifestyle regularity linked to sleep quality [40] and mental health [41]. These outcomes may serve as the pathways towards potentially lower medication use. Relatedly, the gain of new social roles, such as that of becoming a grandparent, or as a volunteer, may evoke new barriers, should individuals perceive or experience such new roles as incompatible with misuse of medication.

## The case of psychoactive prescription drug use in later life

The social context regarding psychoactive prescription substances has changed dramatically recently. The early 21^st^ century was marked by considerable increases in the prescribing of psychoactive substances [42]. Inappropriate prescribing practices have been implicated as a possible cause [43], with some doctors lamenting that it is simply easier to write a prescription than to question a patient’s account of their symptoms [44]. Relative to illicit drugs, research shows that prescription drug use becomes normalized for individuals since such drugs are provided by medical professionals for legitimate purposes [33]. The use of psychoactive medications is particularly common among those in later life. Table 1 shows data from the 2018 National Survey on Drug Use and Health (NSDUH). Regardless of the intended reasons for use, those 50 and older are the most likely to have used a psychoactive (or psychotherapeutic in NSDUH parlance) prescription drug in the past year in total and across several drug classes, including those for pain relief (specifically opioids in the NSDUH) and anxiety (benzodiazepines known as tranquilizers and sedatives) [45].

**Table 1.** Past Year Psychotherapeutic Prescription Use by Age, National Survey on Drug Use and Health 2018

Age	All psychotherapeutic prescriptions	Pain relievers (i.e. opioids)	Tranquilizers	Sedatives
12-17	23.3%	16.8%	3.8%	2.0%
18-25	38.0%	26.7%	10.8%	3.0%
25-34	41.0%	31.2%	12.9%	4.4%
35-49	41.0%	32.9%	14.2%	6.0%
50+	45.2%	36.1%	17.4%	8.7%

*Source*: U.S. Department of Health and Human Services (2020). *N* = 67,791.

In some ways, the fact that older individuals receive more prescriptions for pain and even depression and anxiety than other age categories is not surprising. As noted above, the diagnostic factors that would lead to such prescriptions are a regular part of aging. However, understanding the conditions in which use results from factors other than those indicative of the underlying issue for which the medication is prescribed is important. Given that social loss can be perceived as pain [22], it is possible that individuals could perceive of pain medications as capable of treating other perceived “pains,” such as loss or loneliness that would result from common life course transitions in later life into widowhood, divorce, or retirement, particularly as opioids became commonplace as a legitimate treatment for physical pain. Similarly, depression reaches its heights in older adulthood, and is compounded by personal and status losses [20], and thus depression medication may be perceived as ameliorating such losses. In other words, above and beyond the symptomatic and diagnosable experiences of pain and depression, prescription substances may be used to alleviate feelings that results from common, nonmedical circumstances in later life.

While prescribed for legitimate reasons, prescription pain, depression, and anxiety medication can still lead to adverse outcomes. Along with use generally, prescription drug misuse has also increased among older individuals.^1^ According to the NSDUH, the rate of prescription drug misuse among those 50 and older more than doubled from 2002 to 2016 [45]. This increase is part of a larger overall increasing trend in substance use among older individuals [12, 13], although the case of prescription drug use remains understudied. Mortality in later life has also been affected by psychoactive prescription drugs. According to CDC data, the overdose death rate per 100,000 among those 50 and older increased by over 7 times for prescription opioids and over 10 times for benzodiazepines. The rise in overdose deaths has occurred across all age categories, including in later life, lowering life expectancy across demographic categories [47]. We note that these deaths are recorded as overdoses regardless of the motivation for use, even if the prescription belongs to the user and is used for its intended purpose. Even among patients with a valid prescription, opioid and benzodiazepine dependence and overdose are more likely as dosage and days supply increase [16, 48, 49]. We do not intend to conflate use with these more adverse outcomes such as misuse and mortality. Further, while we look at pain and depression medication use broadly, not all such medications are habit-forming. However, these trends provide a backdrop for our study.

### Current study

We draw on data from the Health and Retirement Study, a rich longitudinal dataset, and social control theory, to examine whether changes in social roles (in family relationships, employment, or civic engagement) may have implications for changes in the use of prescribed pain and depression medications. Further, we examine whether any association persists after adjusting for reported levels of pain and depressive symptoms, as a way towards understanding whether such changes may be linked to physical versus social factors. This may be especially important and novel as existing evidence suggests events such as widowhood and union dissolution, labor market exits, civic engagement, and grandparenthood have independent associations with health and wellbeing outcomes [9, 50–52]. Nevertheless, by adjusting for reported levels of pain and depression across time points, we are able to disaggregate medication usage due to potential changes in pain and depressive symptoms across life course transitions [53], as compared with changes in social role, environment, and context. Understanding and addressing the social context of substance use may also be especially important, as it could help us understand instances when usage may be due to factors beyond experienced and reported pain or depressive symptoms, providing knowledge of events and social contexts that may render individuals more vulnerable to substance misuse.

## Methods

### Data

Our data come from the Health and Retirement Study (HRS), a nationally representative biennial survey of U.S. adults aged 51 and older. Since 1992, the HRS has used a multistage, clustered area probability frame that included an oversample of Black and Hispanic Americans. By 2010, the HRS had included samples from five more birth cohorts: Asset and Health Dynamics among the oldest (AHEAD), Children of the Depression Age (CODA), the War Babies (WB), the Early Baby Boomers (EBB), and the Mid Baby Boomers (MBB).

We note several inclusion criteria for our analyses. First, we use five rounds from years 2006 to 2014, as these are the years in which our dependent variables were queried. All cohorts contributed data in each of these rounds, with the exception of MBB whose data begins when they age into the sample in 2010. Second, the HRS samples an individual in the household based on their age. If that individual has a spouse, that person is also interviewed regardless of their age and continues with the survey even in the case of widowhood or marital dissolution. As we are interested in later life transitions, if the spouse does not belong to one of the above cohorts (i.e. they are younger than the cutoff for MBB), they are not included in our analysis. Third, as several of our variables require answers directly from the respondent, we do not include surveys taken by proxies (6.3% of observations).

### Measures

In a series of questions, respondents are asked if they regularly take prescription medications, from which we derive two dependent variables for prescription medications. Our first dependent variable is the response to whether the respondent regularly takes prescription medication for pain in their joints or muscles. Although respondents are not provided a list of what constitutes prescription pain or depression medications, we briefly describe the classes of substances typical of these medications. Collectively known as analgesics, prescribed pain medications usually fall into two drug classes, both of which are commonly prescribed for older adults [54]: opioids and nonsteroidal anti-inflammatory drugs (NSAIDs). Prescription opioids include hydrocodone (Vicodin), oxycodone (OxyContin, Percocet), morphine, and methadone. Non-opioid prescriptions include stronger versions of over-the-counter drugs such as ibuprofen, naproxen, and aspirin, as well as numerous prescription only NSAIDs. Our second dependent variable is the response to whether the respondent regularly takes prescription medication to help relieve depression or anxiety (we use depression as shorthand below). Unlike pain medications, there are limited over-the-counter options for depression and anxiety, and many medications are used for both afflictions since they are often comorbid and have many of the same symptoms. While there are many classes of prescription depression and anxiety medications, common examples prescribed to older adults include the aforementioned benzodiazepines [55], as well as selective serotonin reuptake inhibitors (SSRIs) and selective serotonin and norepinephrine inhibitors (SNRIs) [56].^2^

For our research question, we believe that the manner in which these question are asked is beneficial. Simply having a prescription would not be enough to answer yes. Rather, the respondent must actually take the medication with regularity. Additionally, by not specifying whether the prescription was for the respondent or whether they follow the instructions as prescribed, the question is inclusive of both intended use and instructions, as well as that which could be defined as misuse.

As we would expect regularly taking medication for pain or depression to be linked to diagnostic measures of these two conditions, our first set of independent variables are meant to tap into measures of pain and depression, respectively. Pain level considers a respondent’s current typical level of pain [58]. Respondents are first asked whether they are “often troubled with pain.” Those who respond “yes” are then asked, “How bad is the pain most of the time: mild, moderate, or severe?” We used these two questions to create a nominal measure of pain level with four categories: none (baseline), mild, moderate, and severe. Depression and anxiety is measured through the Center for Epidemiological Studies Depression (CESD) short-form scale [58–60]. The CESD score is the sum of six “negative” indicators and two “positive” indicators. The negative indicators measure whether the respondent experienced the following sentiments all or most of the time over the past week: depression, everything is an effort, sleep is restless, felt alone, felt sad, and could not get going. The positive indicators measure whether the respondent felt happy and enjoyed life all or most of the time over the past week and are reverse coded. Thus, the higher the score (ranging 0 to 8), the more negative the respondent’s feelings in the past week. Although this variable is constructed by survey administrators, we confirmed its high reliability among the components (alpha = 0.81). Given the possible high endorsement of certain items in the CESD by those reporting pain, we fit several alternative models for regularly taking pain prescription medication with alternate codings and inclusion of CESD, finding similar results. These alternative specifications are described and shown in Supplementary Material A.

Our second set of independent variables includes life course measures for relevant roles in later life. First, marital status is a nominal variable with four categories: married or partnered (baseline), widowed, never married, and separated, divorced, or absent partner. Second, employment status is a nominal variable with three categories: working (baseline), unemployed, and out of the labor force.^3^ Third, volunteering is a nominal variable with five categories representing the number of hours volunteering in the past year: none (baseline), 1-49, 50-99, 100-199, 200 or more.^4^ While our tables show the specific comparison to the baseline categories noted in parentheses above, given that we are interested in all possible transitions, we describe additional pairwise comparisons among the other categories when such comparisons are statistically significant. Finally, we include the respondent’s number of grandchildren. While we included number due to how common it is to have any grandchildren at this stage of the life course, a dummy for any grandchildren leads to the same results (not shown; available upon request).

We recognize that for pain and depression or anxiety, measures of pain and depression are self-reported, even in the clinical setting. Thus, we include numerous time-varying control variables that may be related to regularly taking prescription medications for pain or depression. Continuous variables include the number of doctor visits in the last two years, activities of daily living (ADL; ranging from 0-5 as the sum of difficulties bathing, eating, dressing, walking across a room, and getting in or out of bed), instrumental activities of daily living (IADL; ranging from 0-3 as the sum of using the telephone, taking medication, and managing money),^5^ self-rated health (five categories for poor, fair, good, very good, and excellent), the sum of the number of a list of health conditions (among high blood pressure, diabetes, cancer, lung disease, heart disease, stroke, psychiatric problems, and arthritis), number of living children, number of living siblings, and age in years. Categorical measures include whether the respondent has health insurance (no (baseline) vs. yes), a dichotomy for living in an urban (baseline) or rural area, and whether the respondent currently receives help for ADL or IADL issues or would be able to call on anyone for help in the future if they encountered ADL or IADL issues (no vs. yes). Although we concentrate on the match between the perceived measures of pain and depression and the respective medication outcome, the other measure is included as a control in the model for the other phenomenon (i.e. pain level in the prescription depression medication model and CESD in the prescription pain medication model). As we describe next, our modeling procedure nets out the effect of any time-invariant respondent characteristics, effectively controlling for both measurable (e.g. gender, race/ethnicity, education, cohort) and unmeasurable static factors. However, we note that we also test for whether our reported effects differ by gender (female, male), race/ethnicity (non-Hispanic Black, Hispanic, non-Hispanic White, non-Hispanic Other), and class as measured by education (less than high school, high school, some college, Bachelors or higher). To determine if the timing of the transitions matter, we also test whether our effects differ by age, as measured both by time-varying age and static baseline age in 2006.

### Statistical analysis

We used fixed-effects logistic regression models to determine the effect of our independent variables on regularly taking prescription medication for pain or depression [62, 63]. The method’s strength is the elimination of unobserved heterogeneity by differencing all predictors and the outcome in a given wave from its respondent-specific average for that variable. Fixed-effects estimators are robust to any observed or unobserved time-invariant omitted variables, which removes any constant person-level effects. The interpretation of a fixed-effects model as a within-person effect represents precisely the interpretation we are seeking for our research question. We are interested in whether respondents are more likely to regularly take prescription pain or depression medications in years in which they are in a particular life course status relative to years in a different status, above and beyond any changes occurring in the perceived measures for pain and depression as well as changes in the control variables. For example, are respondents more likely to regularly take pain medications in years when they are widowed compared to years when they are married, controlling for changes in pain level? Given that we are interested in whether current medication use is related to current status compared to medication use in years in a different status, our research question justifies contemporaneous examination rather than any lagged effects.

Additionally, while static characteristics cannot bias estimates, time-varying measures could still differ across static characteristics, which can be tested through an interaction. Thus, to determine if the effects of the life course transitions and indicators of pain and depression differ by gender, race/ethnicity, or class, we tested whether models with these interactions improved model fit via likelihood ratio tests. We note that there is no evidence of issues with collinearity, as the mean (1.31) and highest (1.44) variance inflation factor is well within acceptable limits. Finally, although couples within a single household create possible statistical dependency, fixed-effects logistic regression models are incompatible with cluster-corrected standard errors that would account for this dependency. We fit analogous fixed-effects linear probability models that permit a cluster correction for household and found identical results, confirming the robustness of our findings. These models are shown in Supplementary Material B. We note that these results can be interpreted as marginal effects.

For both prescription medication use outcomes, we present a series of seven models. The first five models show the bivariate effect of the associated diagnostic measure and the four life course variables in order to establish if there is a zero-order relationship (net of fixed effects for respondents). The sixth model then includes these variables in a single model to determine if any significant relationship between the life course transitions and prescription medication use can be explained by the diagnostic measures. Finally, a seventh model adds the described control variables to further rule out any spurious relationships.

As is well-known, fixed-effects models include only those respondents who experienced change on the dependent variable.^6^ For our inclusion criteria, there are 23,447 respondents and 81,694 observations. For pain medication, 27.4 percent of respondents experienced change, such that there are 6,587 respondents and 27,385 observations. After accounting for missing data, the pain medication models include 6,178 respondents and 25,100 observations. For depression medication, 15.3 percent of respondents experienced change, such that there are 3,681 respondents and 15,130 observations. After accounting for missing data, the depression medication models include 3,433 respondents and 13,784 observations. Thus, for both outcomes, few observations are lost to missing data in our models.

## Results

### Descriptive statistics

Table 2 displays descriptive statistics for the sample overall and those observations included in our models (i.e. respondents that experienced change in the outcomes). Across the observations in the HRS, respondents report regularly taking prescription pain medications and depression or anxiety medications 22.9% and 17.8% of the time, respectively. As is not surprising, the percentages are higher when limited to only respondents who experience change on these measures. In the pain model, respondents report regularly taking prescription pain medications in 44.0% of observations. For depression, respondents report taking prescription medications in 45.5% of observations. Those in these subsets also report somewhat higher pain levels and depression scores. Married or partnered and out of the labor force are the most common marital and employment statuses, respectively, regardless of subset. However, those in the model subsets are more likely to be out of the labor force. They are also slightly more likely to be in a marital status other than married or partnered. Overall and across subsets, about a third of respondents spent some time volunteering in the past years, while average number of grandchildren is between four and five.^7^

**Table 2.** Weighted Descriptive Statistics across All Observations, Percentage or Mean (S.D.)

Variable	Overall	Pain Model Subset	Depression Model Subset
Regularly take prescription pain medication	22.9%	44.0%	
Regularly take prescription depression or anxiety medication	17.8%		45.5%
Pain level			
None	64.8%	47.3%	51.0%
Mild	10.3%	14.2%	10.8%
Moderate	19.1%	30.4%	27.8%
Severe	5.7%	8.1%	10.4%
CESD depression score	1.409 (1.978)	1.811 (2.165)	2.165 (2.334)
Marital status			
Married/partnered	64.1%	61.4%	57.6%
Separated/divorced/absent	15.1%	15.3%	18.2%
Widowed	14.9%	18.0%	17.9%
Never married	5.9%	5.3%	6.2%
Employment status			
Employed	44.6%	33.5%	34.4%
Unemployed	2.5%	2.3%	2.3%
Out of labor force	52.9%	64.3%	63.3%
Volunteering in past 12 months			
None	62.6%	66.5%	67.3%
1-49 hours	13.4%	12.3%	12.1%
50-99 hours	8.6%	7.4%	7.7%
100-199 hours	8.4%	7.6%	7.4%
200+ hours	7.0%	6.2%	5.5%
Number of grandchildren	4.408 (5.131)	5.153 (5.486)	4.801 (5.282)
Number of doctor visits in last 2 years	10.494 (20.428)	13.095 (21.342)	13.916 (24.912)
ADL	0.266 (0.784)	0.390 (0.906)	0.456 (1.021)
IADL	0.101 (0.397)	0.144 (0.467)	0.182 (0.529)
Self-rated health	2.772 (1.083)	3.107 (1.034)	3.111 (1.095)
Number of health conditions	2.013 (1.473)	2.591 (1.388)	2.583 (1.523)
Has health insurance	63.6%	57.2%	58.2%
Rural residence	30.2%	31.6%	33.0%
Number of living children	2.867 (1.946)	3.022 (1.974)	2.895 (1.965)
Number of living siblings	2.778 (2.348)	2.591 (1.388)	2.789 (2.374)
Any source of help for/if ADL/IADL issues	68.3%	70.9%	70.6%
Age	66.224 (10.050)	68.188 (10.033)	66.766 (10.010)
Gender: Female	55.3%	59.0%	65.8%
Race/Ethnicity			
Non-Hispanic White	79.5%	76.4%	80.6%
Non-Hispanic Black	9.6%	11.8%	8.2%
Hispanic	7.9%	9.2%	8.4%
Non-Hispanic Other	3.0%	2.6%	2.9%
Education			
Less than high school	16.1%	20.5%	18.9%
High school diploma	31.3%	33.4%	32.9%
Some college	24.4%	24.5%	24.1%
Bachelors or higher	28.3%	21.7%	24.0%
Observations	81,694	25,100	13,784

**Fig. 1.**
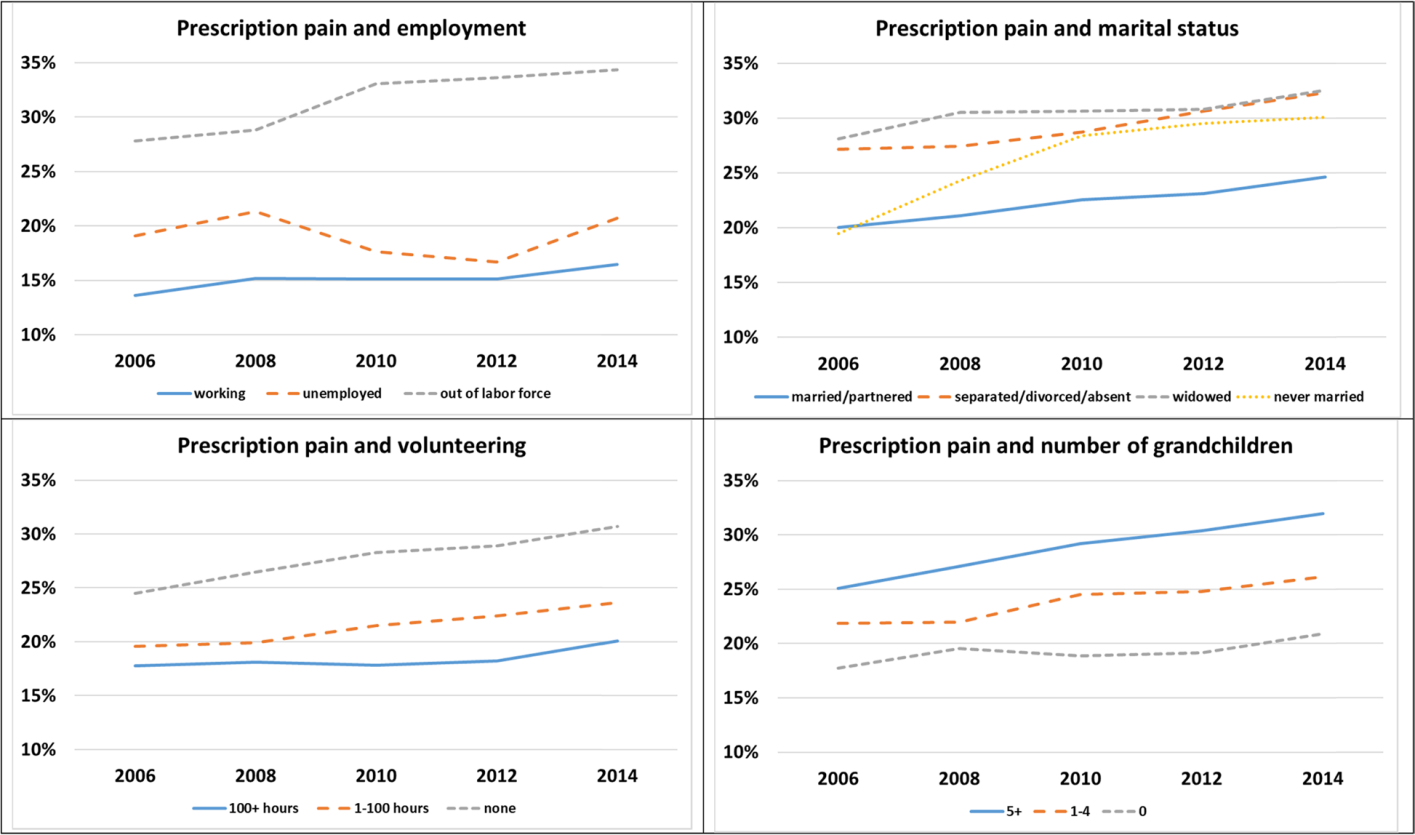
Percentage Regularly Taking Prescription Pain Medications by Life Course Statuses over Time (*N* = 81,694)

**Fig. 2.**
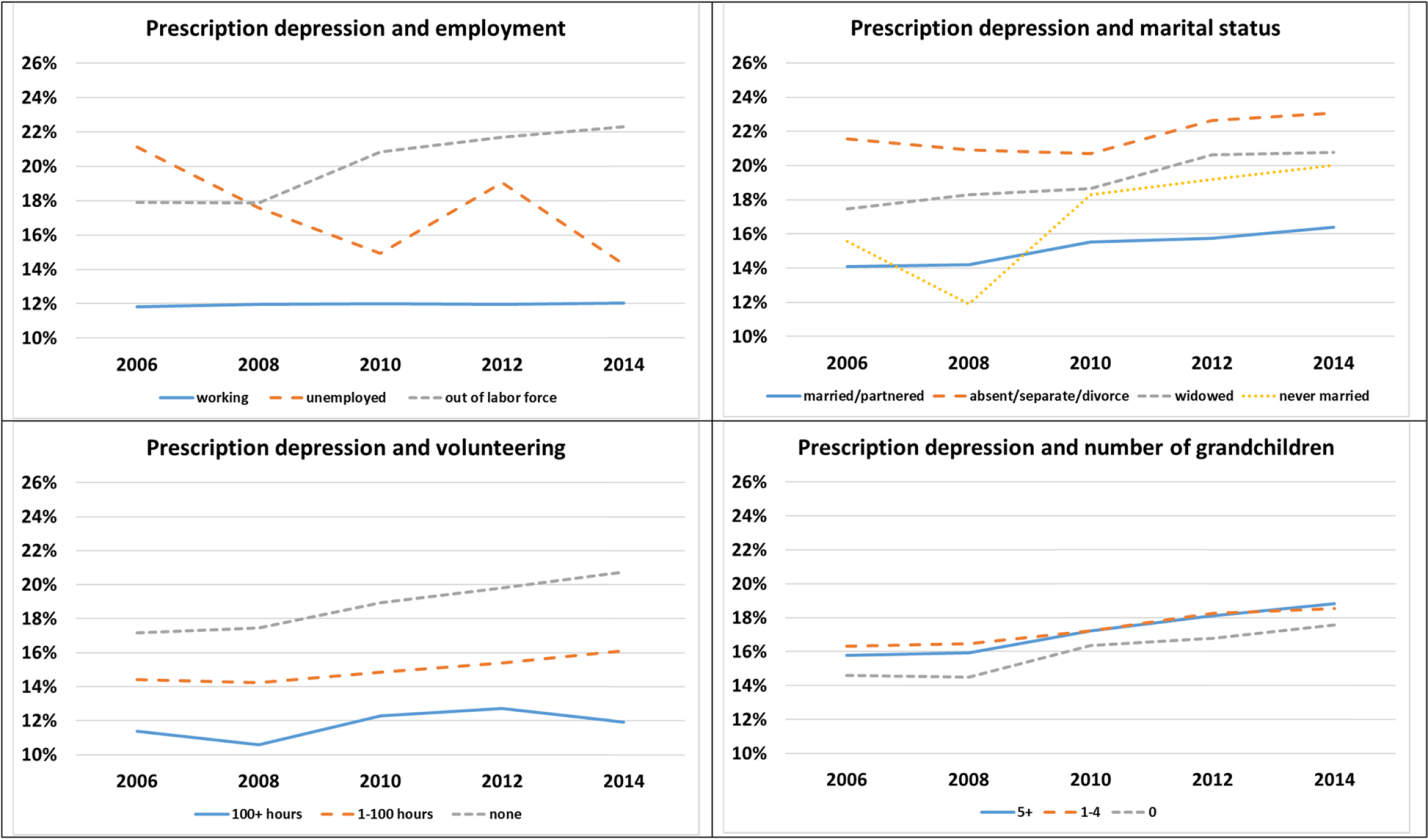
Percentage Regularly Taking Prescription Depression Medications by Life Course Statuses over Time (*N* = 81,694)

Figures 1 and 2 display the observation-level bivariate relationship between the life course roles and our two outcomes of regular use of pain and depression medications, respectively. At least descriptively and with the exception of grandparenthood, there is a noticeable gap between the statuses reflecting being in a role, namely employed, married or partnered, and volunteering, and the other categories reflecting the absence or loss of that status. That is, the percentage reporting taking either prescription pain or depression medications is higher for observations in which respondents report being out of the labor force, widowed or separated, divorced, or partner absent, and no volunteer work. Those with grandchildren actually report taking pain medications more often, with only a very small gap for depression medications. While there is a general increase across the period for all statuses, this trend may reflect changes that occur due to aging. Thus, we turn to our models that attempt to isolate the effect of these life course transitions.

### Fixed effects models

Table 3 displays the results for regularly taking prescription pain medication. Model 1 considers only the perceived measure of pain. The results are as anticipated. Relative to rounds in which the respondent reports no pain, the odds of regularly taking prescription pain medication are 2.2 times higher in years reporting mild pain, 3.1 times higher in years reporting moderate pain, and 5.1 times higher in years reporting severe pain (*p*<.001). We note that all possible pairwise comparisons are also significant at *p*<.001. Considering the other one category increases for example, the odds of regular pain medication use are 42.8 percent higher in years reporting moderate pain relative to years reporting mild pain ([*e*^ln(3.115)-ln(2.182)^ – 1]*100% = 42.8%) and 65.3 percent higher in years reporting severe pain relative to years reporting moderate pain ([*e*^ln(5.148)-ln(3.115)^ – 1]*100% = 65.3%).

**Table 3.** Fixed effects logistic regression for regularly taking prescription pain medication

	Model 1 OR (95% CI)	Model 2 OR (95% CI)	Model 3 OR (95% CI)	Model 4 OR (95% CI)	Model 5 OR (95% CI)	Model 6 OR (95% CI)	Model 7 OR (95% CI)
Pain level (vs. none)							
mild	2.182***					2.148***	1.982***
	(1.982,2.402)					(1.951,2.366)	(1.796,2.187)
moderate	3.115***					3.038***	2.660***
	(2.865,3.387)					(2.793,3.304)	(2.439,2.900)
severe	5.148***					5.017***	4.124***
	(4.498,5.893)					(4.381,5.746)	(3.581,4.749)
Marital (vs married)							
separated/divorced/absent		1.055				1.022	0.825
		(0.872,1.277)				(0.836,1.248)	(0.671,1.015)
widowed		1.320***				1.256**	0.847
		(1.130,1.543)				(1.069,1.476)	(0.714,1.005)
never married		1.013				1.017	0.736
		(0.640,1.605)				(0.632,1.637)	(0.445,1.215)
Employment (vs. employed)							
unemployed			1.005			1.049	0.910
			(0.811,1.246)			(0.840,1.310)	(0.725,1.141)
out of labor force			1.916***			1.733***	1.177**
			(1.722,2.132)			(1.553,1.934)	(1.047,1.322)
Volunteering (vs. none)							
1-49 hours				0.967		0.996	1.067
				(0.872,1.072)		(0.895,1.108)	(0.956,1.190)
50-99 hours				0.843**		0.877	0.957
				(0.741,0.958)		(0.768,1.001)	(0.836,1.096)
100-199 hours				0.810**		0.826**	0.931
				(0.707,0.927)		(0.718,0.950)	(0.806,1.074)
200+ hours				0.734***		0.766**	0.908
				(0.619,0.870)		(0.643,0.914)	(0.758,1.087)
# grandchildren					1.025***	1.021**	1.003
					(1.012,1.039)	(1.007,1.036)	(0.988,1.017)
Depression score							1.026*
							(1.005,1.047)
# doctor visits last 2 yrs							1.004***
							(1.002,1.006)
ADL							1.145***
							(1.093,1.199)
IADL							0.970
							(0.897,1.050)
Self-rated health							1.152***
							(1.101,1.206)
# health conditions							1.357***
							(1.273,1.445)
Has health insurance							0.915*
							(0.839,0.998)
Rural (vs. Urban)							1.108
							(0.897,1.369)
# living children							1.012
							(0.943,1.086)
# living siblings							0.963
							(0.896,1.034)
Any source of help							1.117**
							(1.027,1.215)
Age							1.063***
							(1.049,1.077)
Log-likelihood	-8965.268	-9466.752	-9395.419	-9463.884	-9466.329	-8895.130	-8575.661
Model chi-square	1015.6***	12.7**	155.3***	18.4***	13.5***	1155.9***	1794.8***

Exponentiated coefficients; 95% Confidence Intervals in parentheses. Respondents = 6,178; Observations = 25,100.

* *p* < 0.05, ** *p* < 0.01, *** *p* < 0.001

Models 2 through 5 display findings for the life course variables. For marriage (Model 2), the odds of regularly taking prescription pain medication are 32.0 percent higher in years widowed compared to years married or partnered (*p*<.001). For employment (Model 3), relative to years in which an individual is employed, the odds of regularly taking pain medications are 1.9 times higher in years out of the labor force (*p*<.001). For volunteering (Model 4), relative to years with no volunteering, the odds of taking pain medications are 15.7 percent (*p*<.01), 19.0 percent (*p*<.01), and 26.6 percent (*p*<.001) lower in years volunteering 50-99, 100-199, and 200 or more hours, respectively. These three highest categories are also significant different from years volunteering 1-49 hours. Finally, in years with one additional grandchild (Model 5), the odds of taking pain medication regularly are 2.5 percent higher (*p*<.001). When we move to Model 6 that includes the above measures in one model, we find that the life course status variables are still significant predictors of regularly taking prescription pain medication, even controlling for a measure of pain (with the exception of the 50-99 hours volunteering category). The coefficients for the three predictors are similar to the prior models.

Finally, Model 7 adds the battery of control variables. As expected, reported pain levels are still significant and little altered in magnitude. Although of reduced magnitude, the significant comparison for the employment variable remains so, such that transitions out of the labor force are associated with increased odds of regularly taking prescription pain medication, above and beyond reported pain and the control variables. With controls, relative to years in which an individual is employed, the odds of regularly taking pain medications are 17.7 percent higher in years out of the labor force (*p*<.01). However, the effects of widowhood, volunteering, and grandparenthood are now non-significant.

Table 4 shows the results for regularly taking prescription medication to relieve depression or anxiety. Model 8 considers only the diagnostic measure of depression with the CESD score. Relative to years where the score is one unit lower, in years where a respondent’s score is one unit higher, the odds of regularly taking prescription depression medication are 19.4 percent higher (*p*<.001).

**Table 4.** Fixed effects logistic regression for regularly taking prescription depression medication

	Model 8 OR (95% CI)	Model 9 OR (95% CI)	Model 10 OR (95% CI)	Model 11 OR (95% CI)	Model 12 OR (95% CI)	Model 13 OR (95% CI)	Model 14 OR (95% CI)
Depression score	1.194***					1.176***	1.136***
	(1.168,1.222)					(1.149,1.203)	(1.109,1.164)
Marital (vs married)							
separated/divorced/absent		1.705***				1.536***	1.413**
		(1.346,2.160)				(1.207,1.955)	(1.092,1.829)
widowed		2.312***				1.934***	1.503***
		(1.897,2.818)				(1.578,2.369)	(1.211,1.865)
never married		2.622***				2.482**	2.101*
		(1.518,4.530)				(1.434,4.296)	(1.186,3.723)
Employment (vs. employed)							
unemployed			1.250			1.178	1.060
			(0.938,1.666)			(0.878,1.581)	(0.780,1.439)
out of labor force			1.955***			1.771***	1.234**
			(1.692,2.258)			(1.529,2.051)	(1.054,1.445)
Volunteering (vs. none)							
1-49 hours				0.843*		0.861*	0.919
				(0.732,0.971)		(0.745,0.994)	(0.792,1.066)
50-99 hours				0.714***		0.750**	0.840
				(0.599,0.850)		(0.627,0.896)	(0.699,1.010)
100-199 hours				0.640***		0.667***	0.765**
				(0.532,0.770)		(0.553,0.805)	(0.631,0.929)
200+ hours				0.562***		0.612***	0.728*
				(0.445,0.710)		(0.482,0.777)	(0.569,0.930)
# grandchildren					1.031**	1.030**	1.007
					(1.012,1.052)	(1.010,1.051)	(0.987,1.027)
Pain level (vs. none)							
mild							1.061
							(0.914,1.233)
moderate							1.286***
							(1.136,1.456)
severe							1.521***
							(1.266,1.826)
# doctor visits last 2 yrs							1.005***
							(1.002,1.007)
ADL							1.053
							(0.993,1.118)
IADL							1.289***
							(1.171,1.419)
Self-rated health							1.169***
							(1.099,1.244)
# health conditions							1.668***
							(1.536,1.811)
Has health insurance							1.108
							(0.984,1.249)
Rural (vs. Urban)							0.932
							(0.704,1.233)
# living children							1.122*
							(1.018,1.237)
# living siblings							1.100*
							(1.004,1.205)
Any source of help							1.157**
							(1.036,1.293)
Age							1.014
							(0.995,1.032)
Log-likelihood	-5059.228	-5143.132	-5139.812	-5163.971	-5177.756	-4983.166	-4716.885
Model chi-square	247.0***	79.2***	85.8***	37.5***	9.9**	399.1***	931.6***

Exponentiated coefficients; 95% Confidence Intervals in parentheses. Respondents = 3,433; Observations = 13,784.

* *p* < 0.05, ** *p* < 0.01, *** *p* < 0.001

Models 9 through 12 display results for the life course variables. Compared to years married or partnered (Model 9), the odds of regularly taking prescription depression medication are 70.5 percent higher in years separated, divorced, or absent, 2.3 times higher in years widowed, and 2.6 times higher in years never married (*p*<.001). For employment (Model 10), relative to years in which an individual is employed, the odds of regularly taking depression medications are about 2.0 times higher in years out of the labor force (*p*<.001). For volunteering (Model 11), relative to years with no volunteering, the odds of taking depression medications are 15.7 percent (*p*<.05), 28.6 percent (*p*<.001), 36.0 percent (*p*<.001), and 43.8 percent (*p*<.001) lower in years volunteering 1-49, 50-99, 100-199, and 200 or more hours, respectively. The two highest categories are also significantly different from years volunteering 1-49 hours. Finally, in years with one additional grandchild (Model 12), the odds of taking depression medication regularly are 3.1 percent higher (*p*<.001). When we move to Model 13 that puts these measures in one model, we find that the life course status variables are still significant predictors of regularly taking prescription depression medication, even controlling for a diagnostic measure of depression. The coefficients for the predictors are similar to the prior models.

Model 14 then adds our control variables. As expected, the CESD scale is still significant with a similar magnitude effect. While the two lowest comparison categories for volunteering and grandchildren became non-significant, the remainder of the life course variables are still significant. Relative to years married, the odds of regularly taking prescription depression medications are 41.3 percent higher in years separated, divorced, or absent (*p*<.01), 50.3 percent higher in years widowed (*p*<.001) and 2.1 times higher in years never married (*p*<.05). For the latter then, transitioning into marriage (the only logical order) is associated with lower odds. While reduced in magnitude with the controls, the employment effect also remains significant. Compared to years employed, the odds of regularly taking depression medications are 23.4 percent higher in years out of the labor force (*p*<.01). Relative to years with no volunteering, the odds of taking depression medications are 23.5 percent (*p*<.01) and 27.2 percent (*p*<.05) lower in years volunteering 100-199 and 200 or more hours, respectively.

### Separate effects by gender, race, class, and age

We tested whether the effects of the life course transitions and measures of pain and depression varied in their effect by gender, race, or class as measured by education by adding interactions among these variables to the final model in Table 3 (Model 7) and 4 (Model 14). In no case did the interactions improve the fit of the model for gender (pain: χ^2^=14.95, df=13, *p*>.05; depression: χ^2^=16.57, df=11, *p*>.05), race/ethnicity (pain: χ^2^=36.33, df=39, *p*>.05; depression: χ^2^=25.65, df=33, *p*>.05), or class (pain: χ^2^=40.95, df=39, *p*>.05; depression: χ^2^=42.47, df=33, *p*>.05). We similarly tested interactions with age to determine if timing of transitions mattered. Again, the interactions did not improve the fit of the model, whether age is measured as a time-varying characteristic (pain: χ^2^=15.92, df=13, *p*>.05; depression: χ^2^=4.80, df=11, *p*>.05) or by static baseline age in 2006 (pain: χ^2^=12.34, df=13, *p*>.05; depression: χ^2^=8.09, df=11, *p*>.05). We can thus conclude that the effects presented above do not differ by these measures.

### Reverse causality

In the above models, we assume that the temporal order is such that within-person changes in life course variables affect changes in regularly taking prescription drugs for pain or depression. As a robustness check on our models, we consider the possibility that regularly taking medications for either pain or depression might result in marital dissolution, job loss or ceasing work, or stopping volunteering. To assess this possibility, we created dichotomous indicators for whether unemployed or out of the labor force followed a survey where employed was reported, whether separation, divorce, or absent spouse were reported following a survey where married or partnered was reported, and whether no volunteering followed a survey where volunteering of any hours was reported. We then included regularly taking prescription pain and depression medications, as well as all the other predictors, as lagged independent variables. In this manner, the models assess whether having regularly taken these medications in the prior round is associated with increased odds of a transition out of employment, marriage, or volunteering. Table 5 displays these models. As the rows for the prescription medications show, there is little evidence of the effect operating in the reverse direction. We note these effects are the same even with the perceived pain level and CESD depression measures removed. The exception is the relationship between regularly taking prescription pain medications and a move from married to separated, divorced, or absent. Relative to prior rounds not regularly taking prescription pain medications, having used pain medications in the prior round is associated with 43.3 percent higher odds of moving from married to separated, divorced, or absent in-between rounds. Although we find little evidence that any of the significant effects we found could actually have the reverse order (and some like causing widowhood or grandchild loss are not possible), there is some evidence that perhaps pain medicine use results in marital dissolution. We caution this finding given the two year gap in measurement.

**Table 5.** Fixed effects logistic regression for transitions from employment, marriage, and volunteering

	*Employed to*				*Married to*		*Volunteering to*	
	Unemployed	Unemployed	Out of LF	Out of LF	Div/sep/absent	Div/sep/absent	None	None
*Lagged IVs*	SLR	SLR	SLR	SLR	SLR	SLR	SLR	SLR
Takes Rx pain med	1.083		0.992		1.433**		0.958	
	(0.745,1.574)		(0.860,1.144)		(1.144,1.796)		(0.851,1.078)	
Takes Rx depression		1.041		0.828		0.797		0.952
med		(0.650,1.668)		(0.684,1.004)		(0.595,1.068)		(0.813,1.116)
Marital (vs married)								
separated/divorced/	0.920	0.914	1.056	1.065			1.091	1.095
absent	(0.506,1.672)	(0.503,1.661)	(0.776,1.438)	(0.782,1.450)			(0.849,1.402)	(0.852,1.407)
widowed	0.622	0.622	0.874	0.882			1.001	1.006
	(0.294,1.313)	(0.295,1.315)	(0.653,1.170)	(0.659,1.181)			(0.809,1.238)	(0.813,1.244)
never married	0.927	0.919	0.970	0.971			0.886	0.888
	(0.226,3.797)	(0.223,3.783)	(0.469,2.005)	(0.469,2.007)			(0.439,1.787)	(0.440,1.793)
Volunteering (vs. none)								
1-49 hours	0.738	0.738	1.056	1.056	0.888	0.876		
	(0.521,1.046)	(0.521,1.044)	(0.915,1.218)	(0.915,1.219)	(0.698,1.131)	(0.688,1.114)		
50-99 hours	0.632*	0.632*	0.940	0.940	0.712*	0.713*		
	(0.409,0.978)	(0.408,0.977)	(0.793,1.114)	(0.793,1.113)	(0.520,0.974)	(0.521,0.975)		
100-199 hours	0.630	0.627	0.798*	0.798*	0.492***	0.501***		
	(0.389,1.021)	(0.388,1.015)	(0.660,0.964)	(0.660,0.964)	(0.353,0.684)	(0.360,0.697)		
200+ hours	0.773	0.774	0.884	0.882	0.376***	0.374***		
	(0.464,1.289)	(0.464,1.290)	(0.707,1.105)	(0.705,1.102)	(0.245,0.577)	(0.244,0.574)		
Employment (vs. employed)								
unemployed					1.093	1.099	1.045	1.048
					(0.636,1.879)	(0.640,1.888)	(0.826,1.323)	(0.828,1.326)
out of LF					1.048	1.071	0.925	0.925
					(0.783,1.403)	(0.800,1.434)	(0.808,1.059)	(0.808,1.059)
# grandchildren	0.979	0.978	1.000	1.000	1.027	1.027	1.008	1.008
	(0.910,1.053)	(0.909,1.052)	(0.975,1.026)	(0.974,1.026)	(0.996,1.060)	(0.995,1.059)	(0.987,1.028)	(0.988,1.029)
Pain level (vs. none)								
mild	1.487*	1.484*	0.869	0.869	1.241	1.277	0.949	0.944
	(1.039,2.129)	(1.039,2.119)	(0.741,1.019)	(0.741,1.019)	(0.962,1.600)	(0.991,1.645)	(0.831,1.083)	(0.827,1.077)
moderate	1.297	1.303	0.980	0.983	1.305*	1.366**	1.038	1.033
	(0.920,1.827)	(0.928,1.831)	(0.849,1.132)	(0.852,1.134)	(1.039,1.641)	(1.089,1.715)	(0.921,1.169)	(0.918,1.163)
severe	0.998	1.007	0.781	0.781	1.096	1.200	1.059	1.050
	(0.523,1.904)	(0.529,1.918)	(0.598,1.019)	(0.598,1.018)	(0.736,1.632)	(0.807,1.784)	(0.861,1.302)	(0.855,1.289)
Depression score	0.952	0.954	0.974	0.976	0.912***	0.918***	0.971*	0.971*
	(0.888,1.021)	(0.890,1.023)	(0.942,1.007)	(0.945,1.009)	(0.871,0.955)	(0.876,0.961)	(0.945,0.998)	(0.945,0.999)
# doctor visits last 2 yrs	0.995	0.995	0.997*	0.997*	0.992*	0.992*	1.000	1.000
	(0.985,1.006)	(0.985,1.006)	(0.994,1.000)	(0.994,1.000)	(0.985,0.998)	(0.986,0.998)	(0.997,1.002)	(0.997,1.002)
ADL	0.688*	0.691*	0.889*	0.891*	0.972	0.981	0.982	0.980
	(0.507,0.935)	(0.509,0.938)	(0.805,0.981)	(0.808,0.984)	(0.831,1.136)	(0.840,1.145)	(0.910,1.059)	(0.908,1.057)
IADL	1.078	1.078	0.712**	0.715**	0.776	0.771	0.881	0.888
	(0.691,1.682)	(0.692,1.682)	(0.581,0.874)	(0.583,0.877)	(0.593,1.015)	(0.590,1.008)	(0.767,1.013)	(0.773,1.020)
Self-rated health	1.104	1.101	1.030	1.031	1.156*	1.158**	0.950	0.950
	(0.941,1.295)	(0.939,1.292)	(0.961,1.103)	(0.962,1.104)	(1.034,1.292)	(1.036,1.294)	(0.898,1.004)	(0.898,1.005)
# health conditions	0.812	0.817	0.876**	0.882**	0.957	0.973	0.967	0.969
	(0.630,1.046)	(0.634,1.054)	(0.796,0.963)	(0.802,0.970)	(0.808,1.133)	(0.822,1.152)	(0.892,1.048)	(0.893,1.051)
Has health insurance	3.326***	3.317***	1.630***	1.632***	1.035	1.043	0.955	0.955
	(2.410,4.588)	(2.404,4.575)	(1.433,1.854)	(1.434,1.856)	(0.850,1.261)	(0.856,1.270)	(0.860,1.060)	(0.861,1.061)
Rural (vs. Urban)	0.261**	0.262**	0.631**	0.632**	1.104	1.178	0.875	0.873
	(0.098,0.696)	(0.098,0.701)	(0.446,0.894)	(0.446,0.895)	(0.614,1.984)	(0.651,2.132)	(0.634,1.208)	(0.633,1.206)
# living children	1.013	1.013	0.934	0.937	1.161	1.159	1.022	1.023
	(0.802,1.280)	(0.802,1.280)	(0.847,1.030)	(0.849,1.033)	(0.989,1.362)	(0.988,1.359)	(0.938,1.113)	(0.939,1.114)
# living siblings	1.188*	1.187*	1.047	1.048	0.955	0.956	1.014	1.014
	(1.007,1.403)	(1.006,1.401)	(0.972,1.128)	(0.973,1.129)	(0.822,1.108)	(0.825,1.106)	(0.943,1.091)	(0.943,1.091)
Any source of help	1.011	1.009	1.005	1.008	0.629***	0.640***	1.116*	1.116*
	(0.766,1.333)	(0.765,1.331)	(0.895,1.128)	(0.898,1.132)	(0.524,0.754)	(0.534,0.768)	(1.014,1.229)	(1.013,1.229)
Age	0.934*	0.932**	1.030**	1.030**	1.057***	1.060***	0.999	0.998
	(0.886,0.984)	(0.884,0.982)	(1.009,1.051)	(1.009,1.051)	(1.023,1.092)	(1.026,1.095)	(0.981,1.016)	(0.981,1.016)
Log-likelihood	-701.61	-703.04	-4207.26	-4205.65	-1579.26	-1583.18	-5968.01	-5964.95
Model chi-square	150.665	150.575	130.584	134.377	128.164	120.321	28.505	28.038
Observations	2215	2219	12134	12135	4702	4702	16715	16705

Exponentiated coefficients; 95% Confidence Intervals in parentheses; LF = labor force; SLR = since last round; Rx = prescription; Sep/div/absent = Separated, divorced, or absent spouse

* *p* < 0.05, ** *p* < 0.01, *** *p* < 0.001

## Discussion

Trends in substance use suggest that this is an increasing concern in later life. Though a number of studies have focused on alcohol use as related to life course transitions, there has been less research on prescription medication use, despite the increase in its use as well as related adverse consequences. Our study builds on and extends existing research on later-life transitions on health and health behaviors to highlight whether and how prescription medication use was sensitive to changes in family, labor market, and volunteering transitions. Importantly, while much research has argued that changes in health behaviors may be due to increased pain and stress associated with different life events, our study was able to adjust for this by drawing on a dataset which also contains measures of pain and depression scores. We find as expected that increased reports of pain and depression scores were associated with increased regular use of medication to address these symptoms. Additionally, a novel finding from our study is that even adjusting for self-reports of pain and depression, life course transitions were associated with higher odds of regular prescription medication use, underscoring that social contexts importantly shape substance use beyond that attributed to reported symptoms. In adjusting for reported levels of pain and depression in our study, we clearly show that while these symptoms are one mechanism driving increased substance use, changes in substance use can also be linked to changes above and beyond this pathway. This finding is of potential interests to medical professionals, family members, and policymakers.

Our findings contribute to the literature on the role of social bonds for health outcomes and substance use specifically. A large body of literature, mostly couched in deviance research, overwhelmingly focuses on how bonds provide social controls for adolescent and young adult substance use, emphasizing the importance of family and work [64]. Our finding regarding the transition from never married to married reducing odds of depression medication use shows that early life course studies about the role of bond formation still apply in later life, although this is a rare transition (only 1.4% of all marriage transitions). Given the point in the life course, however, prior research naturally overlooks common role exits such as widowhood and retirement that occur in later life that could be related to substance use through the same mechanisms of social influence. We show that the loss of these bonds in later life, the overwhelmingly most common direction in our data, is also relevant to life course studies of substance use. In line with expectations from social control theory and the role of social integration, we also find this to operate for employment whereby we observed higher odds of regularly taking depression medication in years out of the labour force, as compared to when employed. The health literature similarly has conceptualized these bonds and social roles as providing constraints, regulating behavior, conferring social support, and providing norms that may be internalized around social roles. When the loss of these bonds and social roles occur, however, the associated constraints, regulation from others, support, and internalized norms likely disappear. These changes might not only create the possibility for substance use, but cause the very emotions that might lead to older individuals turning to these substances in the first place. That is, prescription drugs for pain and depression might be used to deal with the resultant loss or loneliness associated with role transitions, with the individual simultaneously having lost the very bond that would have constrained or regulated such behavior.

However, other forms of social support from family such as children and siblings, friends, community organizations, and even medical professionals could possibly fill this role, potentially preventing reliance on medications to address the negative experiences of exiting spousal and work roles [2, 65, 66]. Our findings regarding the mitigating effects of high intensity volunteer work on depression medication use support this assertion. Volunteering provides a context through which older adults may become embedded in a social environment, similar to the workplace and the household environment. Likely, social actors in this environment would exert social support and control to mitigate medication use as theorized above [67]. The dosage response to the hours of volunteering points to the importance of considering the intensity of the role, with hours of volunteering underscoring its salience and centrality to the individual, and also perhaps corresponding levels of social influence and support. However, we note that this finding was limited to depression medication.

By contrast, grandparenthood does not appear to affect prescription medication use once controlling for other measures. One potential explanation may be again due to variation in the salience and intensity of the role and corresponding impact on daily lives. For example, the activities performed as a grandparent may occur with less regularity. Supporting this assertion, one U.S. study found that the mean contact frequency between grandmothers and grandchildren to be between once a week and once a month, while that for grandfathers and grandchildren to be between once a month and once every few months [68, 69]. This again suggests that the impact of social roles may differ contingent on factors such as intensity and duration in which individuals need to enact these roles.

Studies of substance use, and health generally, in later life have focused overwhelmingly on alcohol and tobacco. Our results add to this literature by shifting the focus to another group of substances that are commonly provided for ailments in later life and are readily available to a large subset of older individuals. As we show, individuals in later life regularly use these prescription medications to potentially address the sorts of pains, depressions, and anxieties due to common life course transitions in later life, above and beyond measurable changes in levels of pain and depression. We particularly find that use of depression and anxiety medication is sensitive to these transitions, including both losses and gains in roles. Of course, the toll of these losses and the benefits of these gains on mental health could result in forms of depression and anxiety not captured by our measure of depression or time-varying control measures. That is, there is a direct connection between these transitions and *mental* health. Aside from increases in pain medication use as individuals shifted out of the labor market, the lack of additional effects for pain medications provide reason for optimism. Individuals are perhaps not perceiving the associated “pains” of transitions as additional *physical* pain in need of addressing through pain medication.

Thus, older individuals may consider the pain and depression that could come with exits from life course roles as legitimate reasons to take prescription medications, even though possibly categorized as misuse. Whether characterized as misuse is not necessarily relevant, as adverse outcomes such as dependence and overdose occur even amongst those with valid prescriptions [16, 48, 49]. To prevent adverse events, we encourage states to continue to pass policies, such as prescription drug monitoring programs, that limit overprescribing of controlled substances and hence the amount of controlled prescription drugs in circulation among older individuals. Such policies might spur cultural shifts in medical practice that could have sustained effects on prescribing. We concede that this is a tight line to walk particularly for this age group. Undoubtedly, there are many older individuals that need prescriptions for pain and depression. However, given the evidence here that factors other than pain and depression are driving individuals to take such substances, efforts should be made to reduce prescribing to levels needed for legitimate medical purposes. For example, in recent years there have been moves towards non-medical interventions such as ‘social prescribing’ in countries such as the UK, whereby individuals are directed towards social activities and support services to improve their health and wellbeing [70]. This underscores the value of social and emotional support in conjunction with pharmacological treatments.

Notably, timing of life course transitions and substance misuse may vary across groups within the U.S. Given that we utilized fixed-effects models focused on intra-individual change, our models netted out many time-invariant factors indicative of race, class, and gender. Yet, the reported effects could still differ by these characteristics; however, we found no evidence to support such differences. We still emphasize the need to consider inequalities. Even though these interaction results do not provide evidence that life course transitions or changes in pain or depression levels affect prescription medication use differently by subgroup, the overall pattern of life events and substance use can still differ. Disparities along lines of race, class, and gender undoubtedly shape the observation, timing, and experiences of life course transitions, given variation in the propensity and circumstances upon which individuals will make those transitions [71, 72]. Further, the increase in prescription drug use was highly racialized, in part due to racial discrimination in healthcare; for example, providers were more willing to believe the pain reports of Whites and to prescribe them an opioid painkiller [73–76]. While this may result in Whites having increased opportunity to rely on prescription medications as life course transitions occur, it may also mean that non-Whites seeking similar relief would have to turn disproportionately to non-prescription substances, either licit or illicit. Future research that is able to explicate the importance and role these difference in life course transitions and substance use may play will be equally important.

While there are many advantages to our approach, we must also note some limitations. First, we do not examine the quality of the social roles, and the experience of the various transitions. Potentially, these factors may moderate the extent to which they have implications for medication use. Nevertheless, the findings from this study points to future direction of research, with future studies examining the salience of these factors. Furthermore, while our study focuses on each social role independently and analyses for each life course transitions on medication use independently, there is also the possibility that social roles interact and life course transitions operate conjointly. While outside the scope of the current paper, it points to a future direction of research.

Second, we recognize that a truly objective measure of either pain or depression and anxiety is not available. Even in the clinical setting, measures of these conditions are based on self-reports utilizing scales, and cutoffs for diagnosis are debated. Thus, our measures are similar to those used to diagnose and treat these conditions, hence controlling for the symptoms that these prescription medications are intended to address. Further, the fixed effects net out an individual’s overall propensity to report pain or depression and anxiety, while also controlling for changes in other covariates that might not be fully captured by the self-reports. Third, although we use a measure of depression via the CESD to control for symptoms of both depression and anxiety, we note that these two conditions are often measured with similar indicators (see, e.g., the Hamilton Anxiety Score), comorbid with one another, and use similar medications in their treatment. Fourth, as the respondents are asked broadly about prescription pain and depression medications, these are not defined and thus could add a degree of imprecision. Further, while opioids for pain and most depression and anxiety medications are psychoactive, NSAIDs for pain management are not, and are usually not habit forming. Thus, a subset of respondents may be regularly taking a pain medication without the same potentially adverse consequences associated with opioids or depression medication. Nonetheless, we still find evidence that regular use of the broader class of pain medications are associated with changes in employment beyond subjective pain, and some respondents are likely taking opioids as reflected in the population statistics introduced in Table 1. Relatedly, the only question on prescription pain medications included in the HRS queries “pain in your joints or muscles”; while likely inclusive of many types of chronic and acute pain in older adults, this specificity may miss other forms of pain for which respondents take prescription medications. Fifth, our outcome variables measure current regular use of these prescription medications. We note that “regular” is a subjective notion left to the respondent, although it likely denotes more than occasional use. A measure of frequency of use would certainly be beneficial, but we are limited by the measures in the HRS.^8^ Despite this, we are confident that this represents the best dataset for studying within-individual patterns of pain and depression prescription drug use among older individuals, but encourage future studies to incorporate finer measures. Further, we cannot distinguish whether labor force exits are voluntary or who initiated marital dissolution, which may differentially affect health [9, 77], including substance use patterns. Finally, while recent shifts in prescription drug use generally and among older individuals make these substances an important topic of study in their own right, we note that that this increase is part of an overall rise in substance use among older individuals. While the HRS does not contain any questions regarding cannabis or other illicit substances, we highly encourage future research into the use of illicit substances among older individuals.

Our study and findings nevertheless have a number of implications. For instance, it suggests that medical practice and professionals should be alert to medication utilization around life events for older adults, with caution for overprescribing and over-utilization. At the same time, it highlights how social integration and embeddedness in a regular social network might be important for older adults as they make various life course transitions, as our findings suggest that isolation, as related to the absence of a partner or exiting the labor market, to be associated with increased medication use. Conducted within the background of increasing trends in pharmaceuticalization, our study begins to highlight how medication usage might be related to physical symptoms, but also contingent on social environments, which are important and relevant to understand but potentially also avenues for intervention.

## Supplementary information

**Additional file 1.**

## Data Availability

The HRS is available through the University of Michigan website. (https://hrs.isr.umich.edu/about)

## Footnotes

1 Misuse is defined as taking a prescription substance for a purpose other than prescribed, more than as instructed, or taking a medication not prescribed to an individual [46].

2 While there are additional classes of prescription depression and anxiety medications, such as tricyclic antidepressants (TCAs) and monoamine oxidase inhibitors (MAOIs), they are not recommended for older adults due to their potential side effects [56]. Benzodiazepines also have increased associated risk factors among older adults such as falls [57], but are still commonly prescribed [55].

3 Out of the labor force includes those who responded either “retired” (46.0%) and “not in the labor force” (6.9%). While the latter group likely represents some individuals, such as homemakers, who never worked, the data clearly shows transitions from working to not in the labor force, such that this is not always the case. Thus, some individuals move from working to not in the labor force, but do not label themselves “retired” possibly due to factors such as disability, having worked in a job that does not formerly offer retirement benefits, or the possibility of future employment. In all models, the two coefficients if these categories are separated are nearly identical (and with no statistically significant difference) and all inferential conclusions are the same. For this reason and given that a move from working to either category represent a similar transition away from a bond with employment, we consider them as one category.

4 The categories are derived from a series of questions that help the respondent calibrate their hours volunteered around anchor points. If they said yes, to volunteering, they are asked whether they would say they volunteer in the past year about 100 hours, less than 100 hours or more. From there, 50 and 200 are subsequently used in a similar question depending on if they said fewer or more than 100. The use of these categories is common in studies of volunteering using the HRS (for an overview, see Butrica et al. 2009 [61]).

5 For additional information on validity and reliability of the measures of ADL and IADL in the HRS, see Wallace and Herzog 1995 [59].

6 We emphasize that this same restriction does not apply to the independent variables in a fixed effects model. That is, those who are static on predictors still contribute to the model. Only change on the outcome is required.

7 We note that the direction of transitions among life course statuses is quite consistent at this point in the life course. For example, of all the transitions between married and widowhood, 90% are in the direction of married to widowhood. Similarly, of all the transitions between working to out of the labor force, 83% are from working to out of the labor force. Finally, we also note that some directions are not possible (any marital status to never married) or exceedingly unlikely (reduction in number of grandchildren).

8 We note that the HRS did field a “Prescription Drug Study” in 2005 and 2007 to study medication changes resulting from the implementation of Medicare Part D. In additional to a plethora of information about all prescription drugs generally, this restricted dataset has an inventory of specific prescriptions that details information such as cost and instructed dosage, length, and frequency. This file contains only a subset of respondents: a random sample of those over 65 in 2007 or those covered by Medicare or Medicaid some time between 2002 and 2004, as well as those who were not participating in the simultaneously occurring Consumption and Activities Mail Survey (eliminating 40% of those who fit the first criterion). This random sample amounted to 5,654 persons, with 4,684 participating. While these data have many uses, they are not well-suited for our analysis given only a subset of respondents participated and who were notably older, more general questions about both psychoactive and non-psychoactive medications, only two years of data collection that precludes detailed analyses of changes for both the key predictors and outcomes, lack of a measure of active use of the substances including behavior not following instructions, and lack of an ability to capture use of medications that do not belong to the respondent.
