# Supplementary material for: Later-life transitions and changes in prescription medication use for pain and depression
Source: BMC Geriatr. 2022 Mar 17;22:222. doi: 10.1186/s12877-022-02921-y (PMC8931979; doi:10.1186/s12877-022-02921-y)
Supplement: Supplementary file 1 — Additional file 1. [file 12877_2022_2921_MOESM1_ESM.docx]

**Supplementary Material A: Alternate codings and modeling of CESD in pain models**

The CESD has some items that are highly endorsed by those with chronic pain, such as items measuring activities are an effort, restless sleep, and inability to get going. Thus, when used in chronic pain populations, there is a need to ensure that the total is not inflated by these “vegetative symptoms” that assume good health other than possible depression. We checked reporting any chronic pain against the 8 components of the CESD. Among the three “vegetative symptoms,” those who reported pain said activities are an effort 39% of the time, sleep was restless 48% of the time, and they could not get going 29% of the time. Those who reported pain also reported the remaining five components of the CESD ranging from 15% to 30% of the time. Thus, there may be some inflation for effort and sleep. In order to rule out that this affected the results for regular use of pain medication, we took several approaches shown in Table A1. First, we fit a model between Table 3’s Model 6 and 7 (Model 6a in Table A1) that added CESD, but no other controls. This intermediate model leads to the same inferential conclusions as Model 6 with virtually identical magnitudes of the coefficients, indicating that CESD alone cannot be the reason for any of the changes between Model 6 and 7. Second, we removed CESD from Model 7 (i.e., a model with all controls except CESD; Model 7a in Table A1). Again, this alternative model leads to all the same inferential conclusions with nearly identical magnitude of coefficients. Third, we created a version of the scale that excludes the three “vegetative symptoms”, thus ranging from 0-5 with higher scores indicating higher depressive symptoms and with a reliability of alpha = 0.81 (the same as the 8-item scale). We then included this scale in Model 7 instead of the 8-item CESD (Model 7b in Table A1). Again, this alternative model leads to all the same inferential conclusions with nearly identical magnitude of coefficients. Thus, the inclusion of the three “vegetative symptoms” does not affect the reported results for regular prescription pain medication use.

Table A1: Fixed effects logistic regression for regularly taking prescription pain medication

|  | Model 6a  OR (95% CI) | Model 7a  OR (95% CI) | Model 7b  OR (95% CI) |
| --- | --- | --- | --- |
| Pain level (vs. none) |  |  |  |
| mild | 2.124*** | 1.991*** | 1.988*** |
|  | (1.929,2.339) | (1.804,2.196) | (1.801,2.193) |
| moderate | 2.991*** | 2.673*** | 2.670*** |
|  | (2.749,3.254) | (2.452,2.915) | (2.449,2.911) |
| severe | 4.855*** | 4.169*** | 4.157*** |
|  | (4.237,5.564) | (3.621,4.799) | (3.611,4.787) |
| Marital (vs married) |  |  |  |
| separated/divorced/absent | 0.995 | 0.836 | 0.829 |
|  | (0.814,1.216) | (0.680,1.028) | (0.674,1.020) |
| widowed | 1.218* | 0.861 | 0.852 |
|  | (1.036,1.432) | (0.726,1.020) | (0.718,1.010) |
| never married | 0.981 | 0.749 | 0.741 |
|  | (0.609,1.579) | (0.453,1.237) | (0.448,1.224) |
| Employment (vs. employed) |  |  |  |
| unemployed | 1.034 | 0.916 | 0.912 |
|  | (0.828,1.292) | (0.730,1.148) | (0.727,1.144) |
| out of labor force | 1.714*** | 1.181** | 1.180** |
|  | (1.535,1.913) | (1.051,1.327) | (1.050,1.326) |
| Volunteering (vs. none) |  |  |  |
| 1-49 hours | 1.001 | 1.065 | 1.066 |
|  | (0.899,1.114) | (0.955,1.188) | (0.955,1.189) |
| 50-99 hours | 0.887 | 0.953 | 0.954 |
|  | (0.777,1.012) | (0.832,1.091) | (0.834,1.093) |
| 100-199 hours | 0.836* | 0.927 | 0.928 |
|  | (0.726,0.961) | (0.803,1.070) | (0.804,1.071) |
| 200+ hours | 0.774** | 0.905 | 0.906 |
|  | (0.649,0.923) | (0.756,1.084) | (0.756,1.084) |
| # grandchildren | 1.001 | 1.065 | 1.066 |
|  | (0.899,1.114) | (0.955,1.188) | (0.955,1.189) |
| Depression score | 1.050*** |  |  |
| *(original coding)* | (1.030,1.071) |  |  |
| Depression score |  |  | 1.017 |
| *(alternate coding)* |  |  | (0.989,1.047) |
| # doctor visits last 2 yrs |  | 1.004*** | 1.004*** |
|  |  | (1.002,1.006) | (1.002,1.006) |
| ADL |  | 1.151*** | 1.149*** |
|  |  | (1.100,1.206) | (1.097,1.203) |
| IADL |  | 0.974 | 0.972 |
|  |  | (0.901,1.054) | (0.899,1.052) |
| Self-rated health |  | 1.162*** | 1.158*** |
|  |  | (1.110,1.215) | (1.107,1.212) |
| # health conditions |  | 1.361*** | 1.359*** |
|  |  | (1.277,1.449) | (1.276,1.448) |
| Has health insurance |  | 0.914* | 0.915* |
|  |  | (0.838,0.997) | (0.839,0.998) |
| Rural (vs. Urban) |  | 1.106 | 1.107 |
|  |  | (0.896,1.367) | (0.896,1.368) |
| # living children |  | 1.011 | 1.011 |
|  |  | (0.942,1.085) | (0.942,1.085) |
| # living siblings |  | 0.963 | 0.963 |
|  |  | (0.896,1.034) | (0.896,1.035) |
| Any source of help |  | 1.115* | 1.116** |
|  |  | (1.026,1.213) | (1.027,1.214) |
| Age |  | 1.062*** | 1.063*** |
|  |  | (1.048,1.076) | (1.049,1.077) |
| Log-likelihood | -8883.042 | -8578.691 | -8577.804 |
| Model chi-square | 1180.1*** | 1788.8*** | 1790.1*** |

Exponentiated coefficients; 95% Confidence Intervals in parentheses. Respondents = 6,178; Observations = 25,100.

* *p* < 0.05, ** *p* < 0.01, *** *p* < 0.001

**Supplementary Material B: Linear Probability Models with Household Cluster-Corrected Standard Errors**

Table B1: Fixed effects linear probability model for regularly taking prescription pain medication, with household cluster-corrected standard errors

|  | Model 1  OR (95% CI) | Model 2  OR (95% CI) | Model 3  OR (95% CI) | | Model 4  OR (95% CI) | | Model 5  OR (95% CI) | | Model 6  OR (95% CI) | | Model 7  OR (95% CI) | | |  |  |
| --- | --- | --- | --- | --- | --- | --- | --- | --- | --- | --- | --- | --- | --- | --- | --- |
| Pain level (vs. none) |  |  |  | |  | |  | |  | |  | | |  |  |
| mild | 0.085*** |  |  | |  | |  | | 0.084*** | | 0.075*** | | |  |  |
|  | (0.074,0.096) |  |  | |  | |  | | (0.073,0.094) | | (0.064,0.085) | | |  |  |
| moderate | 0.143*** |  |  | |  | |  | | 0.141*** | | 0.125*** | | |  |  |
|  | (0.132,0.153) |  |  | |  | |  | | (0.130,0.151) | | (0.115,0.136) | | |  |  |
| severe | 0.216*** |  |  | |  | |  | | 0.214*** | | 0.187*** | | |  |  |
|  | (0.198,0.235) |  |  | |  | |  | | (0.196,0.233) | | (0.169,0.205) | | |  |  |
| Marital (vs married) |  |  |  | |  | |  | |  | |  | | |  |  |
| separated/divorced/absent |  | 0.005 | |  | |  | |  | | 0.002 | | -0.013 | | |  |
|  |  | (-0.014,0.023) | |  | |  | |  | | (-0.016,0.021) | | (-0.031,0.005) | | |  |
| widowed |  | 0.027** | |  | |  | |  | | 0.023** | | -0.009 | | |  |
|  |  | (0.011,0.043) | |  | |  | |  | | (0.008,0.039) | | (-0.025,0.007) | | |  |
| never married |  | 0.000 | |  | |  | |  | | -0.001 | | -0.023 | | |  |
|  |  | (-0.043,0.044) | |  | |  | |  | | (-0.043,0.041) | | (-0.064,0.019) | | |  |
| Employment (vs. employed) |  |  |  | |  | |  | |  | |  | | |  |  |
| unemployed |  |  | -0.001 | | |  | |  | | -0.001 | | -0.010 | | |  |
|  |  |  | (-0.018,0.016) | | |  | |  | | (-0.018,0.016) | | (-0.027,0.007) | | |  |
| out of labor force |  |  | 0.059*** | | |  | |  | | 0.050*** | | 0.018*** | | |  |
|  |  |  | (0.048,0.069) | | |  | |  | | (0.040,0.060) | | (0.008,0.028) | | |  |
| Volunteering (vs. none) |  |  |  | |  | |  | |  | |  | | |  |  |
| 1-49 hours |  |  |  | | -0.003 | | |  | | -0.003 | | | 0.003 | | |
|  |  |  |  | | (-0.012,0.005) | | |  | | (-0.011,0.006) | | | (-0.006,0.011) | | |
| 50-99 hours |  |  |  | | -0.015** | | |  | | -0.013* | | | -0.005 | | |
|  |  |  |  | | (-0.025,-0.004) | | |  | | (-0.023,-0.003) | | | (-0.015,0.005) | | |
| 100-199 hours |  |  |  | | -0.018** | | |  | | -0.016** | | | -0.006 | | |
|  |  |  |  | | (-0.030,-0.007) | | |  | | (-0.028,-0.005) | | | (-0.017,0.005) | | |
| 200+ hours |  |  |  | | -0.025*** | | |  | | -0.022** | | | -0.008 | | |
|  |  |  |  | | (-0.039,-0.011) | | |  | | (-0.036,-0.008) | | | (-0.022,0.005) | | |
| # grandchildren |  |  |  | |  | | 0.003*** | | 0.002** | | 0.003** | | |  |  |
|  |  |  |  | |  | | (0.001,0.004) | | (0.001,0.004) | | (0.001,0.005) | | |  |  |
| Depression score |  |  |  | |  | |  | |  | | 0.000*** | | |  |  |
|  |  |  |  | |  | |  | |  | | (0.000,0.001) | | |  |  |
| # doctor visits last 2 yrs |  |  |  | |  | |  | |  | | 0.025*** | | |  |  |
|  |  |  |  | |  | |  | |  | | (0.019,0.031) | | |  |  |
| ADL |  |  |  | |  | |  | |  | | 0.003 | | |  |  |
|  |  |  |  | |  | |  | |  | | (-0.008,0.013) | | |  |  |
| IADL |  |  |  | |  | |  | |  | | 0.012*** | | |  |  |
|  |  |  |  | |  | |  | |  | | (0.008,0.016) | | |  |  |
| Self-rated health |  |  |  | |  | |  | |  | | 0.035*** | | |  |  |
|  |  |  |  | |  | |  | |  | | (0.029,0.042) | | |  |  |
| # health conditions |  |  |  | |  | |  | |  | | -0.007 | | |  |  |
|  |  |  |  | |  | |  | |  | | (-0.015,0.000) | | |  |  |
| Has health insurance |  |  |  | |  | |  | |  | | 0.012 | | |  |  |
|  |  |  |  | |  | |  | |  | | (-0.008,0.033) | | |  |  |
| Rural (vs. Urban) |  |  |  | |  | |  | |  | | 0.002 | | |  |  |
|  |  |  |  | |  | |  | |  | | (-0.004,0.008) | | |  |  |
| # living children |  |  |  | |  | |  | |  | | -0.001 | | |  |  |
|  |  |  |  | |  | |  | |  | | (-0.006,0.003) | | |  |  |
| # living siblings |  |  |  | |  | |  | |  | | 0.010** | | |  |  |
|  |  |  |  | |  | |  | |  | | (0.003,0.017) | | |  |  |
| Any source of help |  |  |  | |  | |  | |  | | 0.004*** | | |  |  |
|  |  |  |  | |  | |  | |  | | (0.003,0.005) | | |  |  |
| Age |  |  |  | |  | |  | |  | | 0.003** | | |  |  |
|  |  |  |  | |  | |  | |  | | (0.001,0.005) | | |  |  |
| (Constant) | 0.201*** | 0.245*** | 0.215*** | | 0.255*** | | 0.236*** | | 0.159*** | | -0.218*** | | |  |  |
|  | (0.197,0.204) | (0.240,0.250) | (0.209,0.222) | | (0.252,0.258) | | (0.228,0.244) | | (0.147,0.171) | | (-0.293,-0.142) | | |  |  |
| Log-likelihood | -1818.639 | -2875.187 | -2771.984 | | -2872.573 | | -2873.007 | | -1702.265 | | -1108.883 | | |  |  |
| Model *F*-test | 301.752*** | 3.616* | 63.993*** | | 4.401** | | 12.751*** | | 77.349*** | | 60.701*** | | |  |  |

95% Confidence Intervals in parentheses. Respondents = 23,447; Observations = 81,694.

* *p* < 0.05, ** *p* < 0.01, *** *p* < 0.001

Table B2: Fixed effects linear probability model for regularly taking prescription depression medication, with household cluster-corrected standard errors

|  | Model 8  OR (95% CI) | Model 9  OR (95% CI) | Model 10  OR (95% CI) | Model 11  OR (95% CI) | Model 12  OR (95% CI) | Model 13  OR (95% CI) | Model 14  OR (95% CI) |
| --- | --- | --- | --- | --- | --- | --- | --- |
| Depression score | 0.014*** |  |  |  |  | 0.013*** | 0.010*** |
|  | (0.012,0.016) |  |  |  |  | (0.011,0.015) | (0.009,0.012) |
| Marital (vs married) |  |  |  |  |  |  |  |
| separated/divorced/absent |  | 0.032*** |  |  |  | 0.026** | 0.021** |
|  |  | (0.017,0.048) |  |  |  | (0.010,0.041) | (0.005,0.036) |
| widowed |  | 0.051*** |  |  |  | 0.040*** | 0.029*** |
|  |  | (0.038,0.065) |  |  |  | (0.027,0.054) | (0.015,0.042) |
| never married |  | 0.057** |  |  |  | 0.052** | 0.044* |
|  |  | (0.023,0.092) |  |  |  | (0.018,0.086) | (0.010,0.078) |
| Employment (vs. employed) |  |  |  |  |  |  |  |
| unemployed |  |  | 0.009 |  |  | 0.007 | 0.004 |
|  |  |  | (-0.003,0.022) |  |  | (-0.006,0.019) | (-0.008,0.017) |
| out of labor force |  |  | 0.033*** |  |  | 0.029*** | 0.012** |
|  |  |  | (0.025,0.040) |  |  | (0.021,0.036) | (0.004,0.019) |
| Volunteering (vs. none) |  |  |  |  |  |  |  |
| 1-49 hours |  |  |  | -0.009** |  | -0.007* | -0.004 |
|  |  |  |  | (-0.015,-0.002) |  | (-0.013,-0.000) | (-0.011,0.002) |
| 50-99 hours |  |  |  | -0.016*** |  | -0.014*** | -0.009* |
|  |  |  |  | (-0.024,-0.009) |  | (-0.022,-0.006) | (-0.017,-0.002) |
| 100-199 hours |  |  |  | -0.022*** |  | -0.020*** | -0.013** |
|  |  |  |  | (-0.030,-0.013) |  | (-0.028,-0.011) | (-0.022,-0.005) |
| 200+ hours |  |  |  | -0.027*** |  | -0.024*** | -0.016** |
|  |  |  |  | (-0.037,-0.016) |  | (-0.034,-0.014) | (-0.026,-0.006) |
| # grandchildren |  |  |  |  | 0.002** | 0.002** | 0.000 |
|  |  |  |  |  | (0.001,0.003) | (0.001,0.003) | (-0.001,0.002) |
| Pain level (vs. none) |  |  |  |  |  |  |  |
| mild |  |  |  |  |  |  | 0.002 |
|  |  |  |  |  |  |  | (-0.005,0.009) |
| moderate |  |  |  |  |  |  | 0.014*** |
|  |  |  |  |  |  |  | (0.007,0.021) |
| severe |  |  |  |  |  |  | 0.031*** |
|  |  |  |  |  |  |  | (0.017,0.044) |
| # doctor visits last 2 yrs |  |  |  |  |  |  | 0.000*** |
|  |  |  |  |  |  |  | (0.000,0.000) |
| ADL |  |  |  |  |  |  | 0.009*** |
|  |  |  |  |  |  |  | (0.004,0.014) |
| IADL |  |  |  |  |  |  | 0.033*** |
|  |  |  |  |  |  |  | (0.024,0.042) |
| Self-rated health |  |  |  |  |  |  | 0.008*** |
|  |  |  |  |  |  |  | (0.005,0.011) |
| # health conditions |  |  |  |  |  |  | 0.037*** |
|  |  |  |  |  |  |  | (0.032,0.042) |
| Has health insurance |  |  |  |  |  |  | 0.005 |
|  |  |  |  |  |  |  | (-0.001,0.010) |
| Rural (vs. Urban) |  |  |  |  |  |  | -0.001 |
|  |  |  |  |  |  |  | (-0.018,0.016) |
| # living children |  |  |  |  |  |  | 0.006* |
|  |  |  |  |  |  |  | (0.001,0.011) |
| # living siblings |  |  |  |  |  |  | 0.004 |
|  |  |  |  |  |  |  | (-0.000,0.009) |
| Any source of help |  |  |  |  |  |  | 0.007* |
|  |  |  |  |  |  |  | (0.001,0.012) |
| Age |  |  |  |  |  |  | -0.000 |
|  |  |  |  |  |  |  | (-0.001,0.000) |
| (Constant) | 0.149*** | 0.153*** | 0.150*** | 0.176*** | 0.161*** | 0.116*** | 0.015 |
|  | (0.146,0.152) | (0.148,0.157) | (0.145,0.154) | (0.174,0.178) | (0.155,0.167) | (0.107,0.125) | (-0.043,0.072) |
| Log-likelihood | 21897.603 | 21680.270 | 21671.165 | 21635.603 | 21617.144 | 22023.923 | 22679.788 |
| Model *F*-test | 208.050*** | 20.739*** | 36.197*** | 8.743*** | 8.927** | 29.721*** | 26.903*** |

95% Confidence Intervals in parentheses. Respondents = 23,444; Observations = 81,695.

* *p* < 0.05, ** *p* < 0.01, *** *p* < 0.001
